# Influence of Pomelo (*Citrus maxima*) Pericarp Essential Oil on the Physicochemical Properties of HomChaiya Rice (*Oryza sativa* L. cv. HomChaiya) Flour-Derived Edible Films

**DOI:** 10.3390/membranes13040435

**Published:** 2023-04-15

**Authors:** Karthikeyan Venkatachalam, Narin Charoenphun

**Affiliations:** 1 Faculty of Innovative Agriculture and Fishery Establishment Project, Surat Thani Campus, Prince of Songkla University, Makham Tia, Mueang, Surat Thani 84000, Thailand; karthikeyan.v@psu.ac.th; 2 Faculty of Science and Arts, Burapha University Chanthaburi Campus, Khamong, Thamai, Chanthaburi 22170, Thailand

**Keywords:** rice flour, essential oil, pomelo pericarp, edible, active film, physicochemical, pathogens

## Abstract

The food industry is increasingly interested in using active edible packaging to address environmental problems caused by conventional synthetic polymers, such as pollution and degradation. The present study took advantage of this opportunity to develop active edible packaging using Hom-Chaiya rice flour (RF), incorporating pomelo pericarp essential oil (PEO) at varying concentrations (1–3%). Films without PEO were used as controls. Various physicochemical parameters, structural and morphological observations were examined in the tested films. Overall, the results showed that the addition of PEO at varying concentrations significantly improved the qualities of the RF edible films, particularly the film’s yellowness (b*) and total color. Furthermore, RF-PEO films with increased concentrations significantly reduced the film’s roughness and relative crystallinity, while increasing opacity. The total moisture content in the films did not differ, but water activity was significantly reduced in the RF-PEO films. Water vapor barrier properties also improved in the RF-PEO films. In addition, textural properties, including tensile strength and elongation at break, were better in the RF-PEO films compared with the control. Fourier-transform infrared spectroscopy (FTIR) revealed strong bonding between the PEO and RF in the film. Morphological studies showed that the addition of PEO smoothed the film’s surface, and this effect increased with concentration. Overall, the biodegradability of the tested films was effective, despite variations; however, a slight advancement in degradation was found in the control film. Lastly, the antimicrobial properties of the RF-PEO films exhibited excellent inhibitory effects against various pathogens, including *Staphylococcus aureus* (*S. aureus*), *Listeria monocytogenes* (*L. monocytogenes*), *Escherichia coli* (*E. coli*), and *Salmonella typhimurium* (*S. typhimurium*). This study demonstrated that RF and PEO could be an effective combination for developing active edible packaging that delivers desirable functional properties and excellent biodegradability.

## 1. Introduction

Currently, food waste presents a major issue in maintaining the quality and safety of food products, leading to negative impacts on a country’s resources and economic growth. Furthermore, oxidation in food results in decreased quality due to its harmful effects, including a reduction in nutritional value and flavor, increased toxicity, color changes, and altered textural properties. As a result, the food industry’s primary objective is to preserve the quality and wholesomeness of food products to ensure consumer acceptance [1]. Plastic has gained popularity as a material for films and coatings because of its versatile properties. However, it poses considerable environmental concerns since it takes a long time to decompose, leading to pollution and other negative effects on ecosystems. To address these challenges, there is a growing interest in developing affordable, eco-friendly, non-toxic, and biodegradable alternatives derived from natural sources [2]. These sustainable alternatives present a valuable option for replacing conventional plastics. Specifically, using natural ingredients derived from resources such as proteins, lipids, or polysaccharides offers great potential for producing biodegradable films. Key polysaccharide components used in preparing edible films include rice flour or starch, chitosan, pullulan, alginate, carrageenan, modified cellulose, pectin, gellan gum, xanthan gum, and others [3]. Among these polysaccharides, rice flour and starch have emerged as appealing options for creating environmentally friendly films. Historically, polysaccharide-based edible packaging has been employed to preserve the appearance and nutritional value of food products [4]. Edible films possess barrier properties and mechanical strength, which help regulate the transfer of food components and their surrounding environment. This contributes to extending the shelf life of food products while improving their quality and appearance [5]. Rice-based materials can provide similar functional properties to traditional plastic films while offering the added benefit of being biodegradable and environmentally friendly [6]. Incorporating plant extracts, particularly essential oils, into edible films can significantly enhance their functionality. By transforming these films into active edible films, they not only serve as a barrier but also exhibit antioxidant and antimicrobial properties. These properties help to control oxidation, which can cause a decline in food quality, and they effectively inhibit the growth of spoilage-causing and pathogenic microorganisms. This results in extending the shelf life and improving the safety of food products while maintaining their sensory attributes [7].

HomChaiya (*Oryza sativa* L. cv. HomChaiya) is a unique native rice variety originating from Chaiya District, Surat Thani Province in Thailand. The grains are short and exhibit a distinct pale brown color, and when cooked under optimal conditions, they exhibit a sticky, soft texture with a delectable fragrance [8]. HomChaiya rice is highly nutritious, containing 18.82% amylose and 8.66% protein [9]. The grains are rich in vitamins (A, E, B1, and B2), minerals (iron, zinc, and calcium), gamma oryzanol, gamma-aminobutyric acid (GABA), and various phytochemicals [10]. Due to its quality, HomChaiya rice has been utilized as a raw material for developing products such as beer [11], yogurt [12], and ice cream [13]. Pomelo (*Citrus maxima*), a fruit belonging to the Rutaceae family, is the largest citrus fruit in terms of size. It comprises 89% water, 10% carbohydrates, 1% protein, and small amounts of fat and vitamin C, similar to other citrus fruits. The essential oil extracted from pomelo pericarp contains key chemical constituents, including limonene, beta-linalool, neral, beta-myrcene, and nootkatone [14]. Pomelo pericarps are abundant by-products with a fascinating chemical composition. Phytochemicals derived from pomelo pericarp essential oil demonstrate various biological effects, such as antioxidant, anti-inflammatory, antimicrobial, anti-pigmentation, anti-hypertensive, anticoagulant, and cytoprotective activities [15]. Edible films and coatings made from biopolymers have attracted considerable attention due to their ability to prevent food spoilage during handling, transportation, and storage, which helps to extend the shelf life of food products. To the best of our knowledge, no studies have been conducted on the development of HomChaiya-rice-flour-based edible films or on the incorporation of pomelo pericarp essential oil (PEO) into such films to produce functional packaging. Therefore, an interesting approach for developing biodegradable, cost-effective, and environmentally friendly films involves combining rice flour with essential oil extracts from pomelo pericarp. The aim of this research was to study the impact of pomelo pericarp essential oil on the physicochemical properties and antimicrobial activity of HomChaiya rice flour films. Furthermore, the insights gained from this research can add value to HomChaiya rice flour as an agricultural product and to by-products from the pomelo pericarp. These findings can be applied to the production of films for food packaging, catering to the needs of industry and consumers in the future.

## 2. Materials and Methods

### 2.1. Materials and Film Formation

De-husked HomChaiya rice was obtained from a local producer in the Chaiya district of Surat Thani province. Upon arrival at the university laboratory, the rice grains were milled into flour using an electric grain mill. The flour was then manually sifted through a 44-mesh sieve. The resulting HomChaiya rice flour (RF) was collected, stored in an airtight container, and used within a week of milling. The proximate composition of RF was analyzed using the Association of Official Analytical Chemists (AOAC) [16] method, yielding the following results: 75.85% carbohydrate [calculation], 9.78% protein [981.10], 9.01% moisture [925.10], 2.13% fat [922.06], 1.89% fat [922.06], and 1.34% fiber [985.29]. All of the chemicals and reagents used in this study were obtained from Sigma-Aldrich, St. Louis, MO, USA. Pomelo fruit (*Citrus grandis* Osbeck, Khao-nahm-peung variety) was purchased from a local market; then, the pericarp was collected and the essential oil was produced using the extraction method proposed by Zhang et al. [17], to be used in the rice film. Pathogenic bacterial strains, including *S. aureus*, *L. monocytogens*, *E. coli*, and *S. typhimurium*, were acquired from the Department of Medical Sciences, Ministry of Public Health, Nonthaburi, Thailand. All of the media for microbial analysis used in this study were obtained from HiMedia, Mumbai, India.

**Figure 1:**
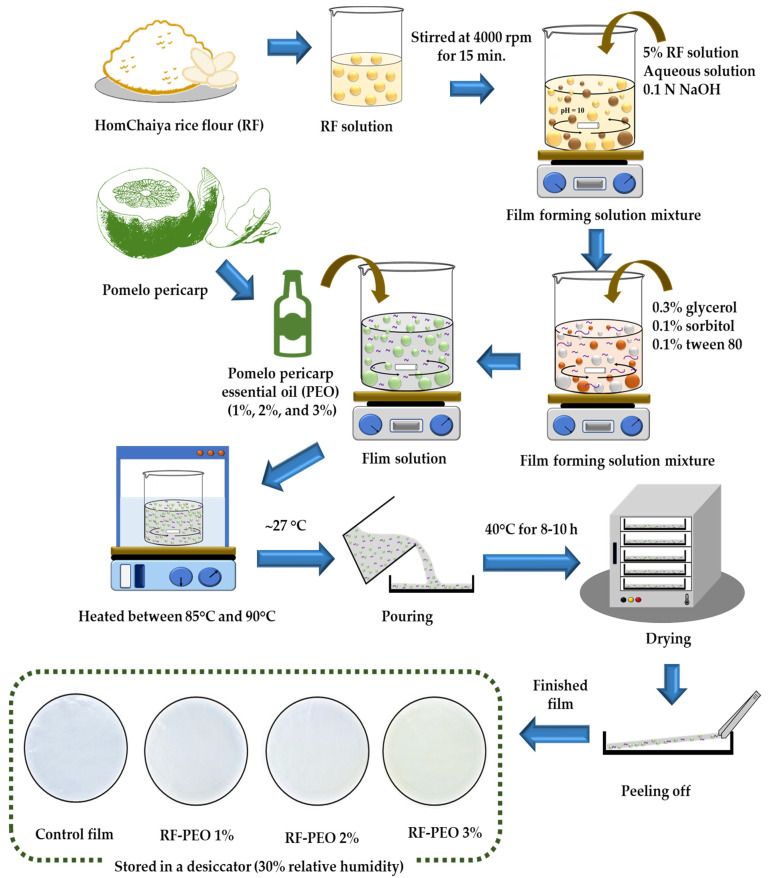
Schematic representation of RF-PEO edible film production.

The RF films were prepared using a casting technique based on the method of Dias et al. [5] with some modifications. To prepare the edible film, 5% RF (5 g) was mixed with distilled water (pH 10, adjusted using 0.1 N NaOH). The mixture was continuously stirred at 4000 rpm for 15 min. Next, plasticizers including 0.3% glycerol (0.3 g), 0.1% sorbitol (0.1 g), and 0.1% Tween 80 (0.1 g) were added to the film-forming solution, followed by the addition of pomelo pericarp essential oil (PEO) at different concentrations (1%, 2%, and 3%, corresponding to 1.0, 2.0, and 3.0 g, respectively). The volume of the distilled water was adjusted to maintain a total volume of 100 mL for each essential oil concentration (94.5 mL for 0% (control), 93.4 mL for PEO 1%, 92.3 mL for PEO 2%, and 91.2 mL for PEO 3% essential oil, respectively). The mixture was then heated to between 85 °C and 90 °C using a thermal water bath and continuously stirred for an hour to initiate starch gelatinization. The temperature of the film-forming solution was brought down to ambient conditions (~27 °C) and poured homogeneously onto plexiglass plates. Then, the film solutions on the plates were dried at 40 °C for 8–10 h in a hot air oven. The films were peeled off of the plexiglass plates and stored in a low-density polyethylene (LDPE)-based Ziplock bag in a desiccator at ambient temperature with 30% relative humidity. The RF films with PEO at different concentrations were designated as treatments and named as follows: RF-PEO 1%, RF-PEO 2%, and RF-PEO 3%. The infographic of the preparation of RF-PEO films is shown in Figure 1. All films in this study were subjected to various determinations as described below.

#### 2.1.1. Optical Properties

The color properties of the examined films, including lightness (L*), redness (a*), and yellowness (b*), were assessed using a Hunter Lab colorimeter (MiniScan EZ, HunterLab, Reston, VA, USA). The total color (ΔE) values of the film samples were measured using the following Equation (1):

(1) $$\mathrm{Toal}\;\mathrm{color}\;\left(\Delta E\right)=\sqrt{{\Delta L}^{*2}+{\Delta a}^{*2}+{\Delta b}^{*2}}$$

In this formula, ΔL*, Δa*, and Δb* represent the differences in the lightness, redness, and yellowness of the color characteristics of the examined films, respectively.

Film roughness was evaluated using atomic force microscopy (diMultitude V, Veeco, Santa Barbara, CA, USA) and NanoScope analysis software (version 1.20, Veeco, Santa Barbara, CA, USA) using the method of Escamilla-Garcia et al. [18]. The opacity of the film samples (5 cm × 2 cm) was examined using a UV spectrophotometer (Shimadzu, UV-1800, Kyoto, Japan). The study was conducted at a wavelength of 600 nm to assess light transmission. The following formula was employed to determine the film’s opacity (2):

(2) $$\mathrm{Opacity}\;\left(\frac{A_{600}}{\mathrm{mm}}\right)=\frac{\mathrm{Absorbance}\;\mathrm{at}\;600\;\mathrm{nm}}{\mathrm{Thickenss}\;\mathrm{of}\;\mathrm{film}\;\left(\mathrm{mm}\right)}$$

#### 2.1.2. Relative Crystallinity

The film samples were measured using an X-ray diffractometer (BRUKER, D2 PHASER, Karlsruhe, Germany). Scan data were collected from a diffraction angle 2θ, ranging from 4 to 50°, in accordance with the method described by Akhila et al. [19]. The TOPAS software (version 5.0, Bruker AXS GmbH, Karlsruhe, Germany, 2012) was employed to compute the relative crystallinity of the films. The formula for estimating the relative crystallinity (3) of the starch granules was as follows:

(3) $$\mathrm{Relative}\;\mathrm{crystallnity}=\frac{\mathrm{Crystalline}\;\mathrm{Area}}{\mathrm{Total}\;\mathrm{area}}\times 100$$

#### 2.1.3. Moisture, Solubility, and Water Vapor Permeability (WVP)

To determine the moisture content, the initial weight of a test film (50 mm × 20 mm) was measured using a four-scale electronic weighing balance. The film was then dried at 105 °C until a constant weight was reached. The moisture content of the film was calculated using Equation (4), with the results presented as percentages.

(4) $$\mathrm{Moisture}\;\mathrm{content}\;\left(\%\right)=\frac{W_{0}-W_{1}}{W_{0}}\times 100$$

In this formula, W_0_ represents the initial weight of a test film, while W_1_ represents the final constant weight of the same test film.

To determine the film solubility in water, film samples were cut into 2 cm × 2 cm pieces, dried at 105 °C for 24 h, and weighed. Each film was placed in a 50 mL beaker containing 20 mL of distilled water, which was sealed and stored at 25 ± 1 °C for 24 h. Afterward, the film pieces were removed and dried at 105 °C for an additional 24 h to determine the final dry matter weight. The results were expressed as percentages.

Water vapor permeability was determined using an adapted gravimetric cup technique. Cylindrical test cups with a diameter of 40 mm were filled with 35 mL of distilled water to maintain 100% relative humidity. Subsequently, the film was applied to the cup, and the cap ensured a tight surface and sealed edges, facilitating water vapor permeation. The initial weight was documented, and the cups were placed in a pre-conditioned desiccator (10–25% relative humidity) with silica gel. The weight of the cups, relative humidity, and temperature were monitored every 2 h for a duration of 24 h. The water vapor permeability (WVP) of the film was computed using a specific Formula (5):

(5) $$\mathrm{WVP}\;\left({\mathrm{Kg}\;\mathrm{Pa}}^{-1}s^{-1}m^{-1}\right)=\left(G\;\times \;X\right)\left[\;t\;\times \;A\;\times \;S\;\times \left(R_{1}-R_{2}\right)\right]$$

In this formula, G denotes the water vapor mass (kg), X symbolizes the film thickness (m), t corresponds to the elapsed time (s), A signifies the area (m^2^), and S represents the saturated water vapor pressure (Pa) at the observed temperature. Additionally, R_1_ refers to the relative humidity inside the cup, while R_2_ stands for the relative humidity in the desiccator.

#### 2.1.4. Thickness and Textural Properties

The film thickness was measured using a handheld digital micrometer (Mitutoyo Series 293 Digimatic Micrometer, Williston, VT, USA), with an accuracy of 0.0025 mm, at six randomly chosen points.

Textural properties, such as tensile strength and elongation at break, were assessed using a texture analyzer. Film samples were prepared by cutting them into 2 cm × 6 cm pieces prior to analysis. Tensile strength was determined at a speed of 0.30 mm/s with a preload of 0.1 N, and the test concluded when the film was torn into two sections. The tensile strength, reported in MPa, was calculated as the ratio of the peak load to the film’s cross-sectional area. Elongation at break was computed as a percentage by dividing the film’s length at the breaking point by its original length (6 cm).

#### 2.1.5. Microstructural Analysis

The microstructure of the films was studied using a scanning electron microscope [20] (JOEL JSM-5410LV, Tokyo, Japan). Prior to analysis, the films were placed in desiccators containing P_2_O_5_ to remove any lingering moisture, achieving a theoretical 0% relative humidity inside the desiccator. The SEM investigation focused on the surface sections of the film samples. To ready the samples for observation, they were fastened to copper stubs, coated with gold for 1.5 min to attain conductivity, and directly viewed at an accelerating voltage of 5 kV and at 1000× magnification.

#### 2.1.6. FTIR

The films were analyzed using FTIR spectroscopy (Thermo Electron Corp., Madison, WI, USA) by following the method described by Lekjing and Venkatachalam [8]. The films were combined with potassium bromide at a 1:100 ratio and subsequently ground into a fine powder before being compressed into a pellet. The analysis aimed to examine potential interactions between rice flour and pomelo pericarp essential oil. Scanning was performed 16 times within a wavelength range of 500–4000 cm^−^^1^ for each spectrum captured.

#### 2.1.7. Biodegradability Test

The process for assessing film sample biodegradation was based on the technique proposed by Oluwasina et al. [21] with some modification. Initially, 2 cm × 2 cm film sections were prepared and dried in a hot air oven for 3 h. Post-drying, the samples were weighed, with the starting weight marked as W_0_. The samples were then positioned in perforated plastic containers and buried at a depth of 3.5 cm in soil. After 24 h, the samples were retrieved, cleaned, and subjected to a second drying phase in a hot air oven at 105 °C for another 3 h. The samples were then re-weighed, and this value was recorded as W_1_. The samples were buried again, with 10 mL of distilled water applied to the soil above them. During the 30-day research period, the samples were removed from the soil every three days, and the weight loss was documented. The biodegradation percentage for each film sample was determined using a specific Formula (6).

(6) $$\mathrm{Biodegradability}\;\left(\%\right)=\frac{\left(W_{0}-W_{1}\right)}{W_{0}}\times 100$$

The film biodegradation was represented as a percentage of weight loss corresponding to the duration of storage days.

#### 2.1.8. Antimicrobial Analysis

The antimicrobial properties of the films were examined using a modified method based on the work of Seydim and Sarikus [22]. Test organisms, including *S. aureus*, *L. monocytogens*, *E. coli*, and *S. typhimurium*, were grown in tryptic soy broth at 37 °C for 24 h. The inhibitory zone test on solid media was utilized to assess the films’ antimicrobial effects. Edible film samples were cut into 10 mm diameter discs, and two discs were gently placed in separate petri dishes filled with a solid medium. For *E. coli* and *S. typhimurium*, tryptic soy agar-based plates were employed, while brain heart infusion agar plates were used for *L. monocytogenes* and *S. aureus*. These plates had been previously inoculated with 0.1 mL of seeding culture. The seeding cultures for all organisms had a concentration of 2 × 10^8^ CFU/mL. After preparing the plates, they were incubated at 37 °C for 24 h in the appropriate chamber. Once the incubation was completed, the plates were examined to identify any inhibition zones surrounding the film discs. The diameters of these zones were measured using a sliding caliper, and the total zone area was subsequently calculated.

#### 2.1.9. Statistical Analysis

Each experiment in this study was performed in triplicate. Data from each analysis were subjected to one-way analysis of variance (ANOVA) and Duncan’s multiple range test (DMRT) using IBM SPSS Statistics software Version 12 (IBM Corporation, Chicago, IL, USA), version 6 for Windows. Experimental data are presented as the mean ± standard deviation (SD), with a significance level of *p* ≤ 0.05.

## 3. Results and Discussion

### 3.1. Optical Properties

**Figure 2:**
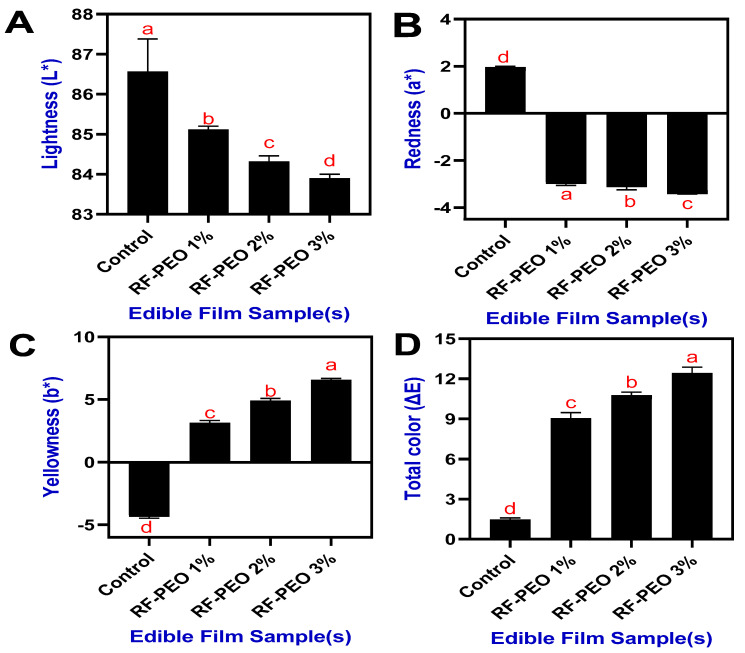
Color characteristics (L* (**A**), a* (**B**), b* (**C**) and ΔE (**D**)) of edible films made of RF-PEO at varying concentrations. The different letters of the alphabet shown on the bar diagram indicate significant differences.

The optical properties, including color characteristics and opacity, of the edible films made from RF and PEO at specific concentrations are shown in Figure 2. Generally, the color and transparency of edible films, which are used as wraps or coatings on food surfaces, play a crucial role in determining the visual appeal and consumer acceptance of food products [23]. This study demonstrated that incorporating essential oil into the RF-based edible film formulations significantly impacted the overall color characteristics of the film and these changes were observed regardless of variations in the PEO concentrations. As the pomelo pericarp ripens, the chlorophyll content (green color) begins to decline while the carotenoid content (yellow color) rises [24]. Therefore, an essential oil extract from pomelo pericarp is a light-yellow liquid with an individual characteristic odor [25].

The film’s color appearance was a pale whitish-yellow, and the addition of PEO to the film composition slightly decreased the lightness and redness values while significantly increasing the yellowness. This is consistent with the findings of Sutput et al. [26], where they discovered that the addition of essential oil significantly increased the yellowness values of the film. Jouki et al. [27] reported that incorporating essential oil into edible film formulations could result in higher positive values of the film, as indicated by increased film yellowness, and subsequently lead to the appearance of a light greenish-yellowish tone. Kong et al. [7] reported that phenolic acid in the PEO influenced the film color characteristics. The total color of the films was also somewhat affected by the PEO concentrations. Higher concentrations of PEO led to increased total color values in the film. 

### 3.2. Film Roughness, Relative Crystallinity, and Opacity

**Figure 3:**
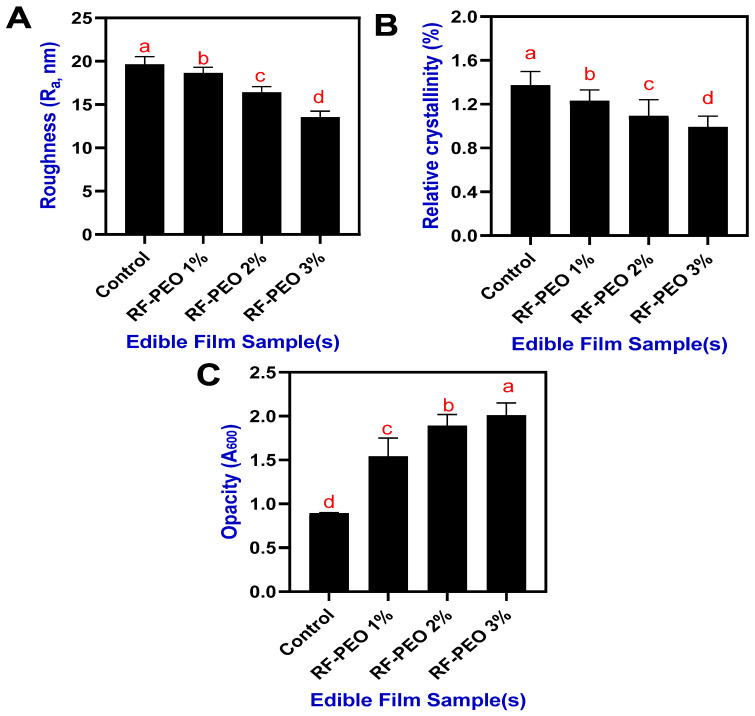
Roughness (**A**), relative crystallinity (**B**), and opacity (**C**) of edible films composed of RF-PEO at varying concentrations. The different letters of the alphabet shown on the bar diagram indicate significant differences.

The roughness of the RF-based edible film incorporating PEO at different concentrations is shown in Figure 3A. Overall, a decrease in roughness was observed in the PEO-added RF films upon increasing concentrations. The control film had the highest roughness (19.64 nm), followed by the RF-PEO films (18.63 to 13.52 nm). In general, the rice-flour- or rice-starch-based edible films exhibit a range of roughness characteristics, specifically, from smooth to rough; this can be controlled by various factors including the raw material source and processing conditions, film preparation method, and the additives and plasticizers used [28,29,30]. The present study showed that the control film had a slightly higher roughness, which could be due to the impaired crosslinking, particularly the covalent and noncovalent bonding between the polysaccharide-based polymers and the additives and plasticizers. However, the addition of PEO in the RF film formation improved the smoothness of the surface. Hosseini et al. [31] also found a similar finding that adding PEO (*Origanum vulgare* L.) to the polymer-based film composition significantly improved the film smoothness.

The addition of EO to the edible film, which contains Tween 80, could positively improve the film surface smoothness by improving the dispersibility and stability of the PEO, and crosslinking [32,33,34]. Similarly, the relative crystallinity of the RF-PEO-based edible films also exhibited a decreasing trend upon increasing concentrations of PEO (Figure 3B). The lowest crystallinity was observed in the RF-PEO 3% film, followed by the others. Furthermore, the control films had a high level of relative crystallinity. Although significant differences were observed among the samples, the overall range was not substantial. The decrease in crystallinity suggests that the addition of PEO interfered with film formation, inducing a plasticizing effect in the RF-based film. This interference disrupted hydrogen bonding within the polysaccharide chain, leading to a reduction in relative crystallinity in the film matrix. These findings align with the study by Jaramillo et al. [35]. Furthermore, the degree of crystallinity in a material can affect its optical properties, including opacity. In general, higher crystallinity can lead to increased opacity due to the more ordered arrangement of the material’s structure, which can scatter light more efficiently. The present study found that when the PEO concentration in the film increased, the level of opacity of the analyzed films decreased significantly (Figure 3C). 

Normally, the opacity value is an important factor to consider when developing or designing food packaging using edible films [36]. This study showed that all the tested edible films in this study exhibited a lower overall range of opacity values. Normally, rice-flour- or rice-starch-based edible films tend to have lower opacity values because the structural integrity of thermally processed rice is weaker. Among the film samples, the control film had the lowest opacity, followed by the RF-PEO film samples, indicating that the addition of essential oil adversely affected the transparency of the film. This phenomenon might be attributed to the presence of essential oil droplets within the film structure, potentially causing enhanced diffuse reflectance due to light scattering. Simona et al. [37] reported that incorporating essential oils into polysaccharide-based edible films resulted in a significant reduction in transparency levels. Similarly, Suptut et al. [26], and Shojabee-Aliabadi et al. [38] found that the phenolic compounds present in essential oils may absorb low-wavelength frequency light on the film surface, leading to decreased transparency in edible films. Furthermore, the amount of amylose content, glycerol, and lipid can alter the opacity level of the starch-based edible films [39]. Normally, the oxidative process in the food product can be altered by controlling the light transfer through the packaging film by enhancing the opacity level by adding essential oil [40].

### 3.3. Moisture, Solubility, and Permeability

**Figure 4:**
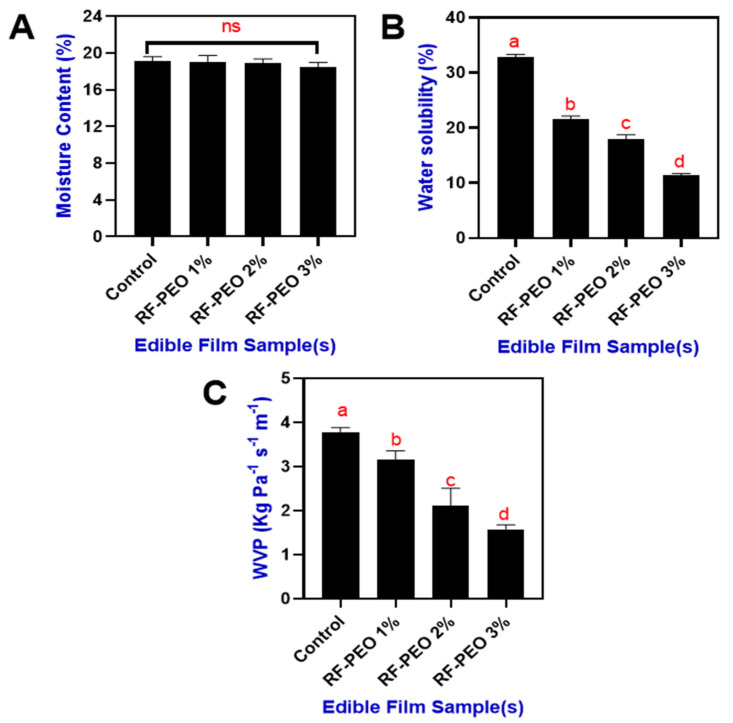
Moisture content (**A**), water solubility (**B**) and WVP (**C**) of edible films that made of RF-PEO at varying concentrations. The different letter of the alphabet shown on the bar diagram indicate significant differences and ns indicates non-significant differences.

The moisture content of the RF-based edible films that incorporated PEO at differing concentrations is shown in Figure 4A. Overall, the moisture content in the film ranged between 19.1 and 18.5% and the incorporation of PEO in the RF edible film slightly decreased the moisture content; however, the differences were not significant. Incorporating essential oil into rice-flour-based edible films may enhance the film’s hydrophobic properties, potentially increasing its resistance to water vapor transmission. Consequently, this improved barrier performance against environmental moisture may result in a reduction in the film’s moisture content. This is in accordance with the study of Song et al. [41], who suggested that the swelling property of the edible film plays a crucial role in its overall moisture content [42]. The study of Socaciu et al. [43] found that addition of essential oil significantly reduced the swelling property of the film. Ojagh et al. [44] reported that integrating essential oil into polysaccharide-based films results in a denser film network. The addition of essential oil encourages covalent bonding between the polymer’s functional groups, diminishing the availability of hydroxyl and amino groups. This mechanism limits polysaccharide–water interactions through hydrogen bonding, consequently reducing the edible film’s moisture content. Furthermore, water solubility is a key factor determining the biodegradability as well as the food applications of edible films made from polysaccharides [7,45]. Water solubility depends on the film’s chemical composition and the interactions between the film’s components and water molecules [46].

Figure 4B presents the water solubility of the tested films. The differences in water solubility between the control and PEO-containing RF films were significant. Among the PEO-containing films, the increasing concentration significantly reduced the water solubility. Water insolubility of the films plays a crucial role in enhancing product integrity and water resistance [47]. The addition of essential oils to a film can influence its water solubility by altering the hydrophilic/hydrophobic balance, interacting with film components, and modifying surface properties. However, the impact depends on the essential oil type and concentration, film composition, and processing conditions [48,49]. In addition, the moisture content and solubility of the film could also affect the water vapor permeability (WVP) properties (Figure 4C). A lower WVP is one of the preferable parameters that accounts for the extension of food shelf life [50]. The incorporation of PEO in the RF films significantly controlled the WVP of the film. Sucheta et al. [51] reported that a lower WVP indicates the homogeneity of the films and holds good potential to be utilized as a packaging material for food applications. A lower WVP in the RF-PEO films may have resulted from the effect of the PEO, which can control water vapor permeability in polysaccharide-based edible films by enhancing hydrophobicity, adjusting film network structure, altering surface properties, and interacting with other film components.

### 3.4. Thickness and Textural Properties

**Figure 5:**
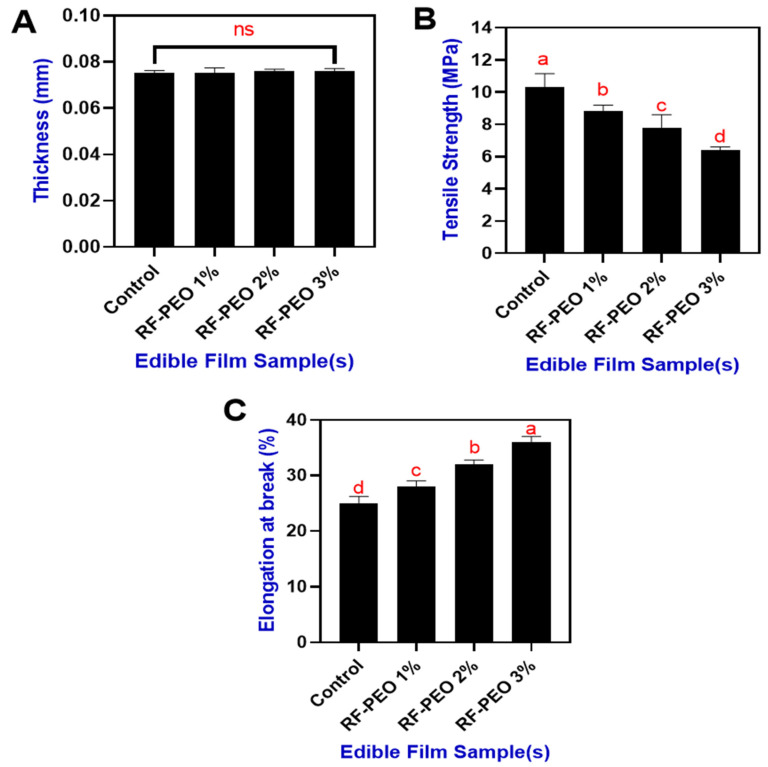
Thickness (**A**), tensile strength (**B**) and elongation at break (**C**) of edible films made of RF-PEO at varying concentrations. The different letters of the alphabet shown on the bar diagram indicate significant differences and ns indicates non-significant differences.

The thickness of the packaging material is crucial for determining the shelf life, biochemical changes, and physical and textural properties of food products [52]. Moreover, Thakur et al. [53] noted that the thickness of a film can impacts its water vapor pressure and transparency. Figure 5A presents the thickness of the edible films made from RF and PEO at specific concentrations. Overall, the film thickness did not differ significantly among samples, and the addition of PEO did not influence the film thickness, despite varying concentrations. The thickness of the RF-PEO-based edible films ranged from 0.0753 to 0.0761 mm. Although a non-significant change in film thickness was observed, the addition of PEO marginally increased the thickness, which could be due to the interaction and physical bonding between PEO and RF. Valizadeh et al. [54] reported that an increase in film thickness is indicative of the formation, interaction, and homogenization of PEO with biopolymers. Several studies have found that increasing rice flour or starch in the film composition increased the thickness of the film. In the present study, the RF concentration was constant, with differences in PEO concentration, and this could provide the rationale for the slight changes in the thickness of the film. Minimal changes in the film made with a fixed starch content concentration may contribute to its swelling power, and the addition of PEO could slightly induce swelling of the film [55]. Mechanical properties of biopolymer films, such as tensile strength and elongation at break, are crucial as they ensure the packaging material maintains sufficient mechanical strength to preserve its integrity throughout handling and storage processes [56].

The tensile strength and elongation at break of the edible films made of RF-PEO at specific concentrations are shown in Figure 5B,C. Overall, this study found that the addition of PEO in the RF-based edible film resulted in a slightly decreased tensile strength and enhanced level of elongation at break. Syafiq et al. [57] reported that incorporating PEO into starch-based polysaccharide polymers negatively affected tensile strength but enhanced elongation at break values. This can be explained by the higher PEO concentrations in the film mixture diminishing the cohesive forces within the polymer chain. As a result, a more heterogeneous matrix is formed, leading to reduced tensile strength and increased elongation. Similar findings have also been reported in various studies by Song et al. [41], Jamroz et al. [58], and Silveira et al. [59].

### 3.5. FTIR Spectrum

**Figure 6:**
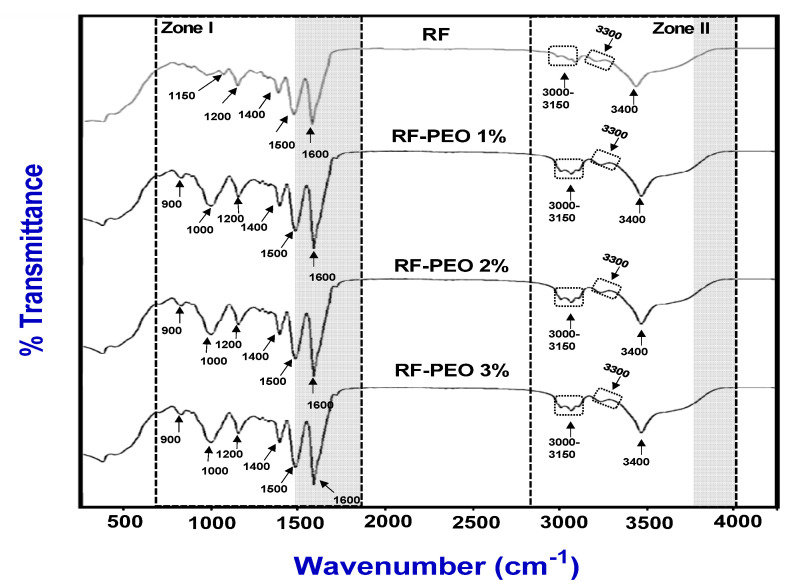
FTIR spectrum of edible films composed of RF-PEO at varying concentrations.

The FTIR spectra of an edible film made of RF and PEO at a specific concentration is presented in Figure 6. In general, the infrared spectroscopy of edible films involves analyzing the absorption of radiation and investigating the manner in which molecules and multi-atomic ions vibrate [60]. The peak pattern analysis revealed that there was a significant difference in peak patterns between the control film and the PEO-containing films. However, no significant differences in peak patterns were observed among the PEO-containing films regardless of the concentration used. Edible films made of starch and non-starch polysaccharides with glycerol as a plasticizer typically display FTIR spectra in the range of 558–2984 cm^−1^, regardless of whether they contain essential-oil-based active ingredients or not [61]. This is in accordance with the observation of present study. This study found major peaks in the FTIR spectra of the tested edible films in two regions: zone I, which ranged from 900 to 1600 cm^−1^, and zone II, which ranged from 2900 to 3400 cm^−1^. Specifically, the FTIR spectra of RF-PEO edible films exhibited characteristic peaks in zone I at specific wavenumbers: 900 cm^−1^ for C-F stretching, 1000–1200 cm^−1^ for C-O stretching, 1400 cm^−1^ for C-F stretching, 1500 cm^−1^ for N-O stretching, and 1600 cm^−1^ for C-C stretching. Conversely, the peaks at 900 and 1000 cm^−1^ were not spotted in the control films. Compared to zone II, the PEO-containing edible films exhibited more noticeable differences than the control samples in zone 1 based on the FTIR spectra peaks. However, the peaks observed in zone II were almost identical in all PEO samples; however, in the control films, the intensity of the peaks ranging between 3000 and 3200 cm^−1^ were slightly low. A decrease in the intensity indicates the lack of PEO in the control film and, consequently, weaker C-H stretching. The FTIR spectra peaks between 3300 and 3400 cm^−1^ represent the stretching vibration of OH and N-H in the samples, respectively. Peaks in this range indicate the stretching vibration of hydroxyl group (OH) and amino group (NH) symmetrical and asymmetrical CH bonds in the polymer structure. This is in accordance with the study of Sihombing et al. [62], Asdagh et al. [60], and Bahram et al. [63]. Furthermore, a reduction in the peak intensities of the hydroxyl group stretching (approximate peaks in the range of 3300–3400 cm^−1^) was noted in both control and RF-PEO edible films. In particular, low peak intensities were observed in the control film compared with the PEO-containing films. This observation suggests that the higher peak intensity in the PEO-containing films is an indication of the presence of crystalline and hydrophobic regions in the film, leading to a higher tensile strength. This is in accordance with the study of Sucheta et al. [51] and Liang and wang [64].

### 3.6. Microstructural Observations

**Figure 7:**
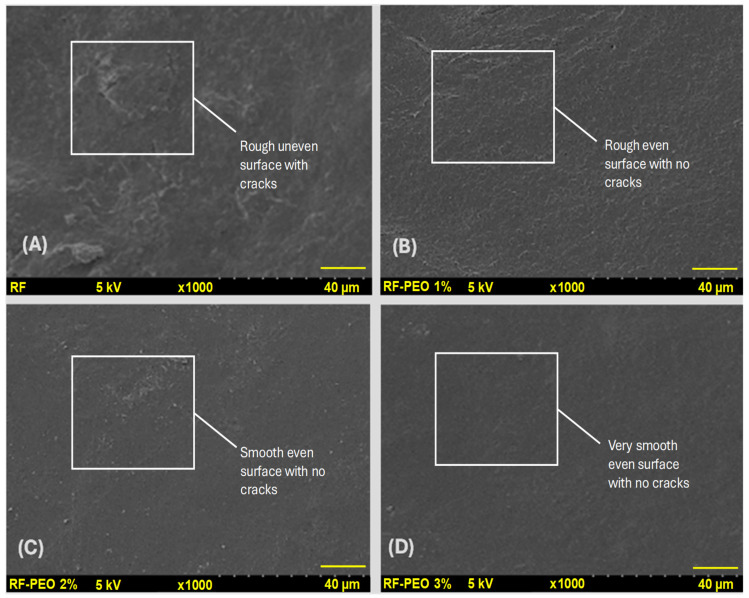
Microstructural observation control film (**A**), RF–PEO 1% (**B**), RF–PEO 2% (**C**), and RF–PEO 3% (**D**) of the edible films composed of RF–PEO at varying concentrations.

Figure 7 depicts the surface microstructure analysis of the edible film composed of RF with varying concentrations of PEO. The microstructural distribution of the edible film is a crucial factor that directly influences the film properties, including physical, optical, mechanical, and barrier properties [20,65,66]. The present study found that microstructural observation indicated a significant improvement in the surface appearance of the RF film upon the addition of PEO compared with the control film. Furthermore, the concentration of PEO had a discernible effect on the film’s appearance, with a higher concentration resulting in a smoother and more cohesive surface, compared with lower PEO concentrations or the control. In contrast to the PEO-containing films, the control film that lacked EO exhibited a rough surface with numerous microcracks. Starch-based films tend to have naturally uneven and rough microstructural surfaces in the absence of optimized additives, which is consistent with the findings of Kang and Song [67] and Acosta et al. [20]. The reason for the uneven surface structure of the edible film may be attributed to the migration of plasticizers to the film’s surface during the drying process, leading to the accumulation of excess plasticizers at the interface [41]. The reason for the smoother surface microstructure observed in the PEO films could be the result of the dispersal effect. This phenomenon occurs when the lipid droplets in the film emulsion create a continuous dispersion in the film’s polymer network, leading to a smoother surface [68]. Syafiq et al. [57] found that the formation of cracks and uneven surfaces on the film could be attributed to the incomplete miscibility of starch in the film emulsion during processing, leading to increased surface coarseness.

Furthermore, a similar effect was discovered on the PEO-containing films, primarily due to the poor compatibility between the PEO and the film polymer that hindered the interconnection of PEO with the film. Consequently, the PEO evaporated from the film during the drying process of the film formation, causing similar surface irregularities. Romani et al. [65] found that a smoother surface microstructure of the edible film could improve mechanical and barrier properties. Their study also suggested that adding PEO to the starch-based edible film helped to produce a continuous polymer matrix phase and improved interfacial interactions. Martins et al. [40] discovered that incorporating PEO into the edible film improved it’s resistance against moisture and water vapor pressure compared with the control film. Their study suggested that this improvement was primarily due to the strong formation of hydrogen bonding between the PEO and the polymer matrix, which enhanced interfacial adhesion and contributed to the desired resistance properties. Zhou et al. [69] observed that incorporating PEO into the starch-based edible film resulted in cracks and holes on the surface due to the coalescence, flocculation, and emulsification of the essential oil during the drying process. This process led to the formation of oil droplets on the film surface, which evaporated quickly due to the high volatility of essential oil, leaving behind holes and cracks. Their study indicated that not all PEOs act as a beneficial additive, and this is solely linked to the interfacial interaction of the PEO and the film. However, the present study revealed that adding PEO to the HomChaiya RF edible film resulted in good structural characteristics indicating that the PEO exhibited better suitability with the tested RF.

### 3.7. Biodegradability

**Figure 8:**
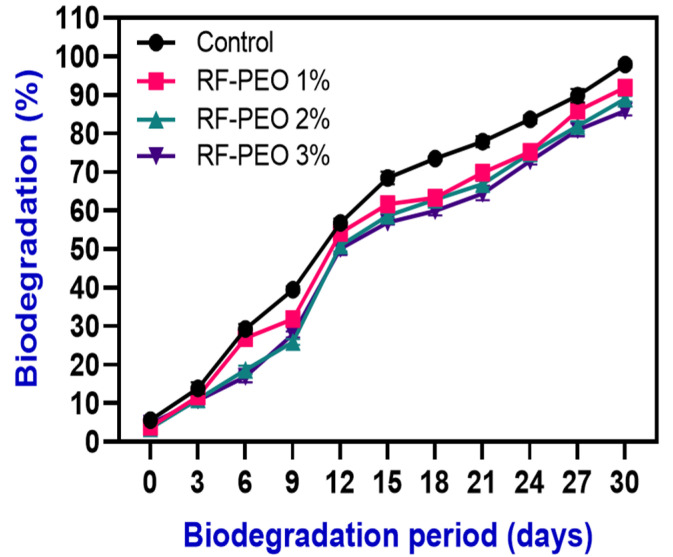
Biodegradability examination of edible films composed of RF–PEO at varying concentrations.

Biodegradability analysis assesses the ability of biodegradable films to break down when exposed to microorganisms in the soil, simulating degradation in a natural environment [70]. The degradation of RF films containing PEO was monitored for 30 days and the results are shown in Figure 8. Within 15 days, over 60% of the samples had degraded, and by 30 days all of the films had fully degraded. The films with lower concentrations of PEO exhibited faster degradation compared with those with higher concentrations. This may be because higher concentrations improved the stability and structural strength of the films and natural plant extracts, particularly essential oils, are known to have strong antimicrobial activity; this could negatively impact biodegradability by inhibiting microbial growth, which is a key factor in biodegradability. This finding is consistent with the study of Mir et al. [71]. Additionally, the hydrophilic nature of the RF film components promotes an increase in water activity, which encourages the growth of microorganisms. According to Dordevic et al. [72], natural polysaccharides, a key ingredient in films, have a more significant role in biodegradation than other types of film-forming materials. Furthermore, the presence of natural fibers in RF that are prone to biodegradation may contribute to the degradation of the film; however, this process is dependent on the degradation of these fibers and the loss of interfacial resistance between the fibers and the polymer matrix.

According to the findings of Suriyatem et al. [73], incorporating plant extracts into starch-based edible films can negatively affect their degradability due to the presence of hydroxyl groups (OH) in the film matrix. A higher level of OH can increase the biodegradability of the film. According to Wittaya [74], various factors such as the amylose and amylopectin content, type and concentration of plasticizers, and lipid content in rice-flour-based edible films can negatively impact their biodegradability. The biodegradability of starch-based edible films is significantly influenced by factors such as the type and moisture content of the soil, microorganisms present in the soil, weather conditions, and various film properties including water vapor pressure, water absorption, and thickness [75]. The biodegradability results of the films indicate that RF films with PEO can be considered to be biodegradable materials that can be returned to the environment without causing harm.

### 3.8. Antimicrobial Activity

**Figure 9:**
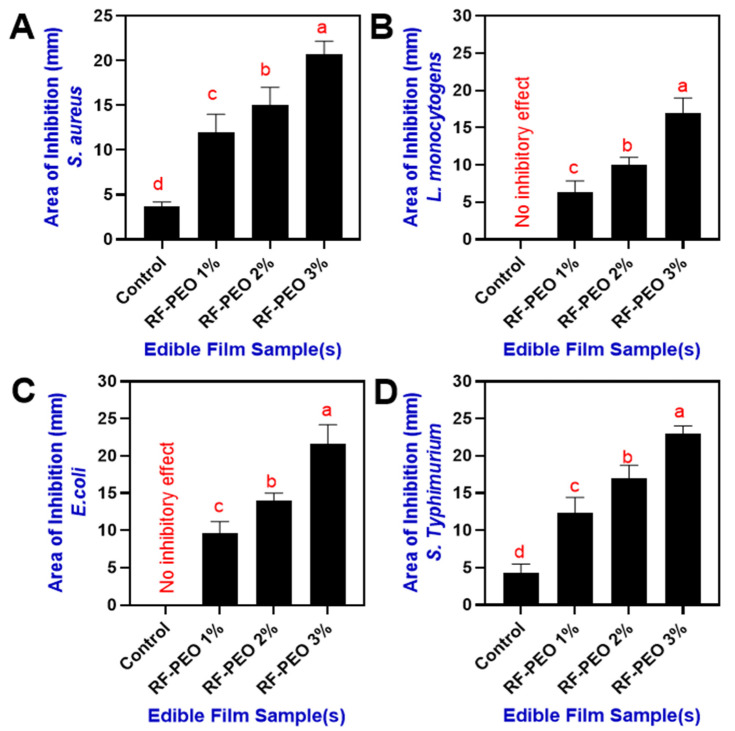
Antimicrobial activity of edible films composed of RF–PEO at varying concentrations against selected pathogens including (**A**) *S. aureus*, (**B**) *L. monocytogens*, (**C**) *E. coli*, and (**D**) *S. typhi-murium*.

Incorporating natural antimicrobial compounds into biopolymer-derived edible films and coatings has greatly improved the effectiveness of active food packaging systems [76]. The antimicrobial activity against the *S. aureus*, *L. monocytogens*, *E. coli*, and *S. typhimurium* by RF films containing PEO at different concentrations are shown in Figure 9. In comparison with the control film, the PEO-containing RF films significantly controlled the growth of the tested microorganisms. Furthermore, the effect of PEO against microbial growth was influenced by its concentration; higher concentrations (>2%) demonstrated a better inhibitory effect compared with the lower concentration and control. The control film was the least effective against microorganisms due to the lack of antimicrobial compounds, and the addition of PEO effectively improved the antimicrobial activity of the film. Chanthaporn et al. [77] investigated the antimicrobial activity of pomelo pericarp extract and its essential oil. Their research revealed that fresh pericarp had a stronger inhibitory effect against *S. aureus*, *L. monocytogenes*, and *E. coli*. They also found that the minimum inhibitory concentration for PEO was >2.25 mg/mL, higher than for other essential oils such as kaffir lime and lime. This aligns with the current study, where PEO combined with the RF film at concentrations above 2% demonstrated improved antimicrobial activity against the tested pathogens. Suklampoo et al. [78] found that the antimicrobial activity of pomelo pericarp extract and its byproducts depends on the fruit variety and pericarp parts. The flavedo extract from the Khao-nahm-peung variety exhibited stronger antimicrobial activity against both Gram-positive and Gram-negative bacteria compared to the albedo part and the Khao-paen fruit variety, which showed minimal or no effect. Palazzolo et al. [79] observed that PEO typically has greater efficacy against Gram-positive bacteria than Gram-negative bacteria. This is consistent with the current study, in which RF-PEO edible films were more effective at reducing *S. aureus* and *L. monocytogenes* than *E. coli* and *Salmonella* species. Barrion et al. [80] noted that the antimicrobial properties of pomelo pericarp are due to its phytochemical composition, specifically tannins, flavonoids, and saponins. Sreepian et al. [81] reported that PEO’s antibacterial effects result from its hydrophobic properties and limonene content, and PEO targets the bacterial components such as membrane fatty acids, proteins, and ATP/ATPases. Their hydrophobicity allows them to infiltrate bacterial membranes, disrupt lipids, and alter permeability, causing cellular content leakage, potassium ion reduction, and hindered respiration.

## 4. Conclusions

The present study demonstrated the feasibility of creating edible films that are biodegradable and gluten-free using HomChaiya rice flour and pomelo pericarp essential oil at different concentrations. The results showed that these films were cohesive, visually uniform, and flexible. The addition of the essential oil resulted in a smoother film that was less transparent, with a distinctive yellowish tint and a stronger pericarp color. The structural properties of the films with essential oil were similar in terms of uniformity, compactness, and molecular structure, except for the control film, which had a rough surface. The essential oil concentration impacted the films’ mechanical properties and water vapor permeability, enhancing the tensile strength and elongation at break. The films containing the essential oil had improved water vapor barrier properties compared with the control film. Additionally, the films with essential oil had a strong inhibitory effect against pathogenic bacteria, which increased with the concentration of oil used. Our findings indicate that pomelo pericarp essential oil at 2–3% can be used as a reinforcing additive in gluten-free rice flour films, making these films a potential source of biodegradable and edible materials.

## Data Availability

All of the data are already included within the article text.
